# Periodontitis

**Published:** 1862-06

**Authors:** G. W. Ellis


PERIODONTITIS.—*By G. W. Ellis, M. D.*—Periodontitis, literally rendered, signifies "inflammation around the tooth." The disease to which this term is applied, owing to the frequency of its occurrence, the great amount of pain attending it, and the readiness with which it generally yields to proper treatment, is one of the most interesting pathological conditions of the dental organ.

But a few years since, when the practice of dentistry was confined almost exclusively to rude and uneducated operators, the treatment and preservation of painful or diseased teeth was a matter never contemplated, and extraction constituting the only reliable means of affording permanent relief, valuable teeth were at once sacrificed without any regard to either their beauty or utility; but thanks to those scientific men through whose untiring efforts dentistry has been led, by rapid advances, to an exalted and enviable position among the sciences, we are now enabled to treat succcssfully almost every abnormal condition of the teeth and their appendages. Periodontitis may be produced by a variety of causes, mostly found among those contained in the following list: 1. The extension of inflammatory action from the pulp through the apicial foramen. 2. Tendency or determination of blood caused by extirpation of the pulp. 3. Caries so located as to impinge upon the cementum, the sharp, uneven edges of the cavity acting as an irritant, or by its walls lodging and holding in contact with the membrane decomposed and irritating substances. 4. Protrusion of fillings upon contiguous parts. 5. Salivary calculi deposited in contact with the periodonteum. 6. Mechanical injury. 7. Imperfect occlusion. 8. Loose fangs. 9. Irritating local applications, such as arsenic, etc. 10. Medicines whose effects are manifested in the teeth and jaws, such as mercury, phosphorus, etc. Inflammation of the dental periosteum is produced by the same general causes, and pursues the same course as inflammation in other tissues, occasioning, however, a class of symptoms somewhat different, owing to the anatomical peculiarities of the parts involved. It has been divided into two classes, the dental and alveolar, according as the membrane covering the root or lining the socket may be affected; this, however, is regarded as rather a useless distinction, for it is doubtful, owing to the close contiguity of these membranes—one, in fact, being but a reflection of the other—whether either one is often diseased without somewhat implicating its neighbor. But it is a matter of indifference which view may be entertained, since its adoption will not have the effect of producing any modification in the treatment.

Periosteal inflammation may be either *acute* or *chronic*. Under the former head are classed those cases in which the inflammation is well marked, the pain severe, and which, without treatment, would terminate in the formation of pus; under the latter head are placed those cases where the inflammation is of the sluggish or asthenic character, occasioning little or no pain, and often running on entirely unnoticed by the patient, producing thickening of the membrane and final loss of the tooth. The type of the inflammation is influenced both by the temperament of the patient and the character of the exciting cause, the acute variety occurring mostly in persons of full or sanguine temperament, and where the exciting cause has been violent and of limited duration; the chronic form being more prevalent in individuals of the lymphatic or bilious temperaments, and where the cause of trouble has been less violent and longer continued. The larger proportion of cases which we are called upon to treat being acute in their character, this form of the disease merits the first and more careful consideration. Commencing our observations with an examination of its symptoms, we notice that the first indication of an attack of acute periodontitis is manifested in feelings of slight uneasiness or tension, accompanied by a desire to work or press upon the affected tooth, for gentle and steady pressure will be found to give relief so long as it is continued; but on its withdrawal, the morbid sensations again return with equal if not increased vigor. But suddenly striking or tapping upon a tooth in this condition will cause great pain, and constitutes one of our most reliable means of diagnosis. Owing to the relief experienced from the application of pressure, we will always observe the patient closing the jaws firmly together so as to bring the painful tooth in contact with the antagonizing one, if such there be; but in consequence of the inability to keep them permanently in this position, the diseased organ is subjected to an alternate application and removal of pressure, serving only to hasten and increase the trouble, clearly indicating, as the first step in the treatment, the protection of the tooth by the introduction of some suitable apparatus to prevent occlusion. In a short time the uneasiness will culminate in a dull, heavy pain; the gum will become marked red, and considerably swollen, particularly in the depression near the root; the tooth will feel as if lengthened, and the parts become so tender that there is no longer any desire to remove it, and the slightest tap upon it will produce so much pain that even closure of the jaws becomes impossible.

If no efforts are made to arrest the progress of the disease, that portion of the periodonteum affected will lose its vitality, become detached from the surface of the cementum, and secrete pus, constituting an affection known as *alveolar abscess.* In cases, however, where the whole of the membrane is implicated and finally destroyed, the tooth, cut off from all connections with the economy, becomes what is termed necrosed or dead, and, being a foreign body, produces the usual effects of such causes upon the surrounding tissues.

The treatment of periodontitis may be either prophylactic or curative, the former being based upon three local and three general considerations, viz.: 1. The removal of all irritating causes, either mechanical or vital. 2. Absolute rest of the part. 3. Applications of a tonic, astringent, sedative, or stimulant nature, as required. The constitutional treatment consists: 1. In the regulation of diet. 2. Bodily rest and quiet. 3. The administration of an aperient. In cases, however, where the acute inflammatory action has become fully established, the usual means for combating it elsewhere must be brought into requisition. Local blood-letting is indicated, not directly over, but on either side of the inflamed part, care being taken to neither hasten nor retard the hemorrhage. The use of the pediluvium as a derivant is advantageous, and the administration of a cathartic with the same view is also of great importance, mercury in some form proving very efficient, a cachectic or scrofulous diathesis, however, always contraindicating its exhibition.

In acute periodontitis great benefit is derived from holding in *constant* contact with the part a mouth-wash, containing some antiphlogistic remedy, such as chlorate of potassa or acetate of lead, the former being strongly recommended in cases of high inflammatory action.

In the treatment of these cases, it is important to bear in mind the beneficial effects which result from maintaining the head in an elevated position, thus causing the blood to gravitate from rather than to the part, where we already have a superabundance. The observance of this fact will often render efforts successful, which might otherwise have proven abortive.

The foregoing summary contains the most reliable and generally adopted treatment of periodontitis. It frequently becomes necessary, however, to combine with it the administration of constitutional remedies, such as arterial sedatives, diaphoretics, etc., which will be found to prove highly efficacious in subduing local inflammation. In this connection, it may be proper to make a few remarks in relation to the treatment of acute periodontitis in teeth where the nerve has been removed, and call attention to a remedy, the use of which, in such cases, suggested itself to me about a year ago, and which has been used ever since, both by Dr. Flagg (to whom I at that time mentioned it) and by myself, with the greatest success—I allude to nitrate of potassa, or nitre, as it is commonly termed.

In my first use of the material, I employed a saturated aqueous solution, applied upon a roll of cotton, carefully introduced well up into the root. By this plan I obtained very satisfactory results; not so much so, however, as by pursuing the method afterward suggested by Dr. Flagg, of using it in the form of a powder, thoroughly packed in the fang canal by means of fang pluggers, thus securing the full antiphlogistic effects of the undiluted article. The fang and pulp cavity should be entirely filled with the material, the remaining cavity being stopped with some substance impervious to the saliva, which will require but little force either in its introduction or removal. On removing the outside stopping, in the course of a day or two the nitre will be found to have entirely disappeared, so that nothing will be necessary, save merely to refill and stop as before. The application should be renewed as often as the judgment of the operator may dictate, care being taken, however, in its introduction, to avoid prying or irritating the diseased tooth. Used in this manner, I think it will prove itself a valuable and reliable remedy.

In chronic inflammation of the dental periosteum the treatment, as in the acute variety, is both prophylactic and curative, the former consisting in the removal of any exciting cause and the employment of stimulant applications; the latter treatment must be conducted slowly, without endeavoring to produce any sudden effects, and consists in the stimulation and toning of the parts, and the application of a sorbefacient, such as iodine, to reduce any existing induration. Sometimes, however, this form of the disease is so far advanced before we are enabled to see the case, that extraction presents the only means of relief.—*Dental Cosmos.*

